# Comparative outcome of different treatment options for fragility fractures of the sacrum

**DOI:** 10.1186/s12891-022-06039-5

**Published:** 2022-12-19

**Authors:** Julian Ramin Andresen, Sebastian Radmer, Reimer Andresen, Axel Prokop, Guido Schröder, Urs Nissen, Hans-Christof Schober

**Affiliations:** 1 Medical School, Sigmund Freud University, Vienna, Austria; 2 Centre for Orthopaedics, Berlin, Germany; 3 Institute of Diagnostic and Interventional Radiology/Neuroradiology, Westküstenklinikum Heide, Academic Teaching Hospital of the Universities of Kiel, Lübeck and Hamburg, Heide, Germany; 4 Department of Trauma Surgery, Sindelfingen, Academic Teaching Hospital of the University of Tübingen, Tübingen, Germany; 5 Clinic of Orthopaedics and Trauma Surgery, Warnow Klinik, Bützow, Germany; 6 Department of Neurosurgery and Spine Surgery, Westküstenklinikum Heide, Academic Teaching Hospital of the Universities of Kiel, Lübeck and Hamburg, Heide, Germany; 7 Department of Internal Medicine IV, Municipal Hospital Südstadt Rostock, Academic Teaching Hospital of the University of Rostock, Rostock, Germany

**Keywords:** Pelvic osteosynthesis, Sacrum, Osteoporosis, Sacral fragility fracture, Sacral insufficiency fracture, Sacroplasty, Pain therapy, Cement augmentation

## Abstract

**Background:**

Fragility fractures of the sacrum (FFS) have been detected more and more frequently in recent times, and the incidence will continue to increase due to increasing life expectancy. The aim of this study was to compare the clinical outcome of conservative, interventional and surgical treatment of FFS.

**Methods:**

Retrospectively, 292 patients (276 women, 16 men) with confirmed FFS were followed up over a period of 2 years. The age of the women was Ø 81.2 (58 - 99) and that of the men Ø 78.1 (76 - 85) years. The pain was quantified using a VAS. Fractures were classified in accordance with the Rommens and Hofmann and with the Denis classification using conventional X-rays, CT and MRI. A QCT of the lumbar spine was performed to quantify bone mineral density. Concomitant diseases of every patient were recorded. An interdisciplinary case conference determined the individual treatment concept considering the age, type of fracture, pain level and comorbidities with classification into conservative, interventional (any type of sacroplasty) or surgical treatment. Over the course pain and independence were measured, complications and patient satisfaction were documented. A vitamin D determination was done, and existing comorbidities were included.

**Results:**

Patients with a pain level of ≤5 benefited from the conservative therapy measures, with pain levels > 5 significantly delaying the development of mobility. After sacroplasty, the pain reduced significantly, which caused a rapid improvement in mobility without any significant difference being found between vertebro- (VSP), balloon (BSP), radiofrequency (RFS) and cement sacroplasty (CSP). In terms of pain reduction and mobilization, the surgical treated patients benefited from osteosynthesis, although more complex fracture types with lumbopelvic stabilization took longer. Overall, there were no deaths during the hospital stay. Mortality after 12 months was 21.7% for the conservative, 8.4% for the interventional and 13.6% for the surgical therapy group; the differences are significant. For patients in the conservative therapy group who were difficult to mobilize due to pain, the mortality increased to 24.3%. Over 24 months, patients achieved the best independence after sacroplasty. At 12 and 24 months, subjective satisfaction with the therapies was best after sacroplasty, followed by osteosynthesis and conservative measures. All patients had a pronounced vitamin D deficiency and manifest osteoporosis. Cardiovascular pathologies were the main concomitant diseases.

**Conclusions:**

Patients with FFS with a low level of pain benefit from conservative therapy measures, whereby complications and mortality increase significantly in the case of persistent immobilizing pain. Patients with an unacceptable level of pain resulting from non-dislocated fractures benefit significantly from sacroplasty. Patients with unstable and displaced fractures (Rommens and Hofmann type III and IV) should be operated on promptly. Different techniques are available for sacroplasty and osteosynthesis, which lead to an improvement of independence and a reduction in mortality.

## Background

Fragility fractures of the sacrum (FFS, synonym: sacral insufficiency fractures, osteoporotic sacral fractures) are increasingly being found in patients with reduced bone quality, rheumatoid arthritis, condition after radiotherapy on the pelvis and after cortisone medication, whereby older postmenopausal women with osteoporosis show the highest risk profile [1–8]. In patients from such risk groups, an incidence of up to around 5% is suspected [9], although precise figures are not available at present. On the basis of demographic development, with the proportion of over 80-year-olds roughly doubling up to the year 2040 [10], a marked increase in FFS is to be expected in the coming years [11, 12]. Since the first description of three osteoporosis patients with sacral insufficiency fractures by Lourie in 1982 [7], doctors have been becoming increasingly aware of this type of fracture as a result of the growing sensitisation for the clinical signs and more targeted imaging diagnostics [11, 13]. The fracture is often the first manifestation site in the pelvis, followed by fractures in the pubic ramus, the parasymphyseal region, the acetabulum and the iliac crest, while conversely 56-90% of FFS are found after previously described fractures in the anterior pelvic ring [14–17]. The fracture develops without or after low-energy trauma in a structurally and substance rarefied bone [18] and is itself an indicator fracture for the presence of clinically manifest osteoporosis [15]. Severe, disabling pain in the lower back, buttocks and groin, as well as local pain upon pressure to the fracture zone are the primary clinical signs [17, 19–21]. Patients are usually unable to stand or walk, with increasing immobilisation in the case of bilateral fracture involvement, whereby neurological deficits are rare and, if present, typically manifest in an isolated S1 syndrome or in a cauda equina syndrome limited to the sacral nerve roots [22].

The aim of the present study was to compare the feasibility and clinical outcome of conservative, interventional and surgical treatment of FFS.

## Methods

### Recording of patients

Patients with tumour-related osseous destruction or pathologic fractures and courses after high-energy trauma were excluded. Retrospectively, 292 patients (276 women, 16 men) with confirmed FFS were followed up over a period of 2 years after therapy, consultation of the patients took place every 6 months. The recruitment period was from January 2014 until June 2019. The age of the women was Ø 81.2 (58 - 99), that of the men Ø 78.1 (76 - 85) years. Immobilizing pain was quantified on a visual analogue scale (VAS) [23].

### Imaging for fracture classification and determination of osteoporosis

On the basis of conventional radiographs (a.p., inlet and outlet images of the pelvis), CT (axial 0.625 mm slice thickness with a 2 mm axial, coronal, semi-coronal slice plane oblique to the sacrum and sagittal reconstruction, with documentation in the bone and soft tissue window) and MRI examinations of the pelvis (with the sequences: T1-, T2-mDIXION axial; STIR semi-coronal oblique to the sacrum and T2 fat-suppressed sagittal; with a respective slice thickness of 4 mm), a classification of Fragility Fractures of the Pelvis (FFP) according to Rommens & Hofmann [14] and categorisation of fractures according to Denis et al. [24] was performed. For osteoporosis diagnosis, a QCT (GE Revolution EVO / 64 line CT, Mindways Software 3D Volumetric QCT Spine) of the lumbar spine was performed. Additional fracture of the axial and peripheral skeleton were also recorded, taking into account X-ray images and medical history.

### Procedure for therapy planning

An interdisciplinary case conference determined the individual treatment concept with classification for conservative, interventional or surgical treatment, taking into account the fracture morphology, pain, concomitant diseases and the will of the patient.

Depending on the intensity of pain, the conservative treatment included bed rest, adjuvant medicinal pain therapy according to the WHO schedule [25] and mobilisation using a walker or on forearm crutches with pain-adapted weight-bearing.

The various different methods available for interventional treatment by means of sacroplasty are described by Andresen et al. [26] and those for surgical treatment using different osteosyntheses are described by Oberkircher et al. [27].

Pain intensity was measured with the VAS score at different time-points after diagnosis, self-reliance was measured by means of a modified Hamburg Barthel Index (HBI, Table 1) [28], while complications including death, and patient satisfaction were recorded.

**Table 1:** Adapted Hamburg Barthel Index according to Luebke et al. [28]

		Score
Feeding	- self-reliant, independent	10
	- requires a little help	5
	- not self-reliant	0
Transfer bed/wheelchair	- independent in all phases	15
	- little help or supervision	10
	- considerable help with transfer and change of position	5
	- not self-reliant	0
Washing	- independent in all phases of activity	5
	- not self-reliant	0
Toilet use	- independent in all phases	10
	- requires help	5
	- not self-reliant	0
Bathing	- independent in taking a bath or shower	5
	- not self-reliant	0
Walking on corridor level or wheelchair use	- independent walking 50 m	15
	- can walk 50 m with walking aids	10
	- not self-reliant, 50 m are possible with a wheelchair	5
	- not self-reliant walking or using a wheelchair	0
Stairs	- independent when climbing stairs	10
	- requires help	5
	- not self-reliant, even with help	0
Dressing and undressing	- independent	10
	- requires help	5
	- not self-reliant, even when help is given	0
Stool control	- completely continent	10
	- occasionally incontinent	5
	- frequently/constantly incontinent	0
Bladder control	- completely continent	10
	- occasionally incontinent	5
	- frequently/constantly incontinent	0
	Total (range 0 – 100)	

Vitamin D was determined in all patients. Any vitamin D deficiency was immediately corrected, and permanent medication was continued according to the DVO guideline [29]; further anti-osteoporotic drug therapy was recommended. Any concomitant diseases present were also recorded.

## Statistics

Statistical analysis of the results was performed using Prism 8 software (GraphPad). The Mann Whitney test was used for unpaired samples to compare the individual therapies and the Wilcoxon rank sum test was used for paired samples to determine changes over time. The students t-test was used to compare means between two groups (bone mineral density (BMD) or vitamin D values between patients with a unilateral and bilateral fracture). For comparisons between individual groups (mortality rate after conventional, interventional and osteosynthetic therapy), the one-factor analysis of variance (ANOVA) was used. At the same time, the effect sizes were calculated according to Cohen and values < 0.5 were assumed to be a small effect, between 0.5 and 0.8 a medium effect and > 0.8 a large effect. Statistical significance was marked as significant = *p* < 0.05, highly significant = *p* < 0.005 and very highly significant = *p* < 0.0005.

## Results

### Fracture classification

**Fig. 1:**
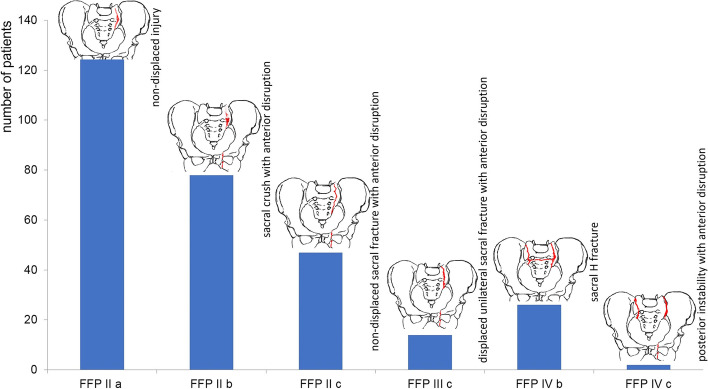
Number of patients with corresponding FFP types according to Rommens & Hofmann [17]. Only FFP types with involvement of an FFS are included; FFP types without a sacral insufficiency fracture were not considered

According to the classification of Rommens & Hofmann, FFP type II with unilateral and/or bilateral fractures (type II a, type II b and type II c) was found in 250 of 292 (85.7%), FFP type III c in 14 of 292 (4.8%) and FFP type IV (type IV b and type IV c) in 28 of 292 (9.5%) of the patients treated (Fig. 1).

Of the 292 patients treated, 91 (31.2%) had unilateral and 201 (68.8%) had bilateral FFS, totalling 493 FFS.

**Fig. 2:**
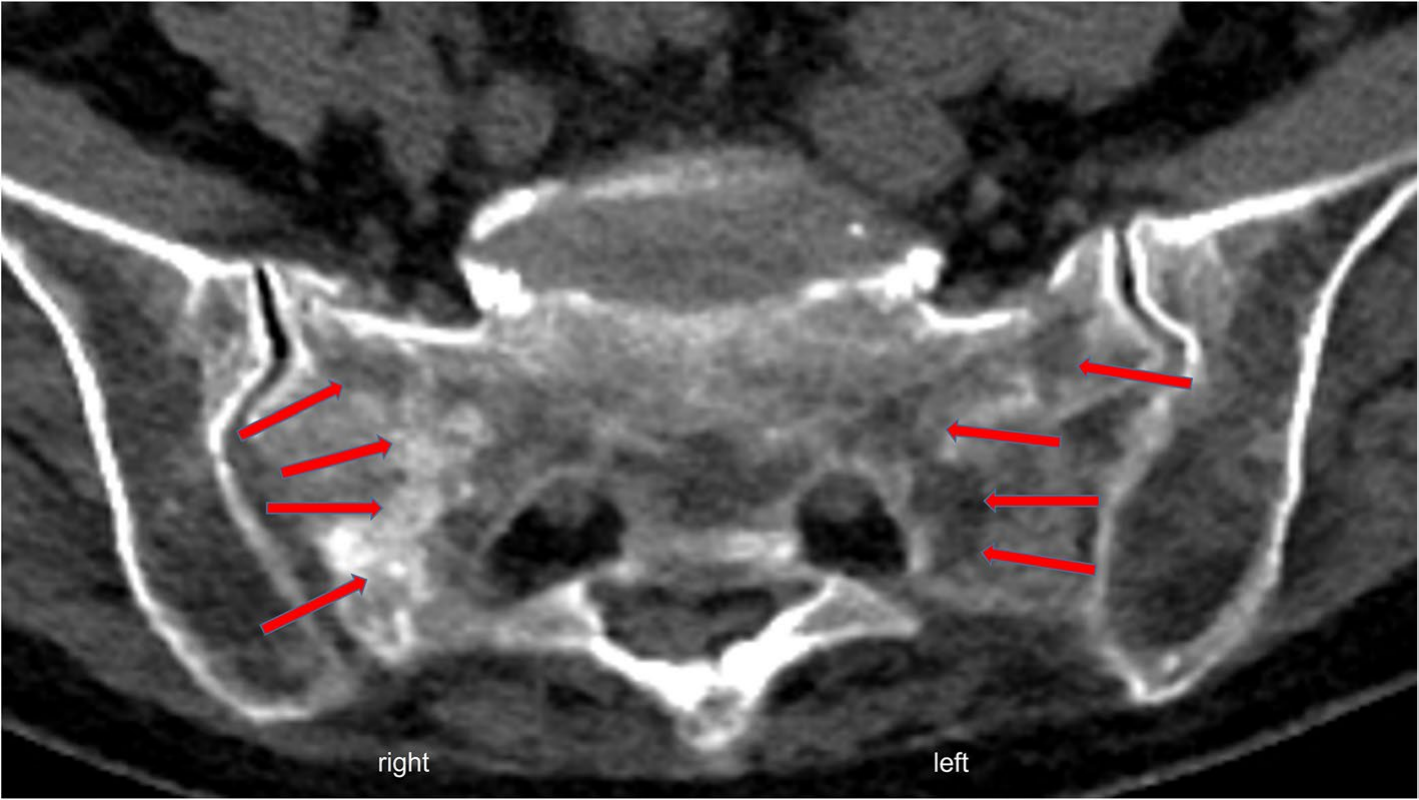
Axial CT slice shows a bilateral FFS of different ages with marked sclerosis in the fracture zone on the right and without remodelling reactions in the fracture zone on the left

As an indication of the different ages of the FFS, the bilateral fractures usually showed varying degrees of oedema and in some cases laterally differentiated sclerosis in the area of the fracture zones on CT and MRI imaging (Fig. 2).

**Fig. 3:**
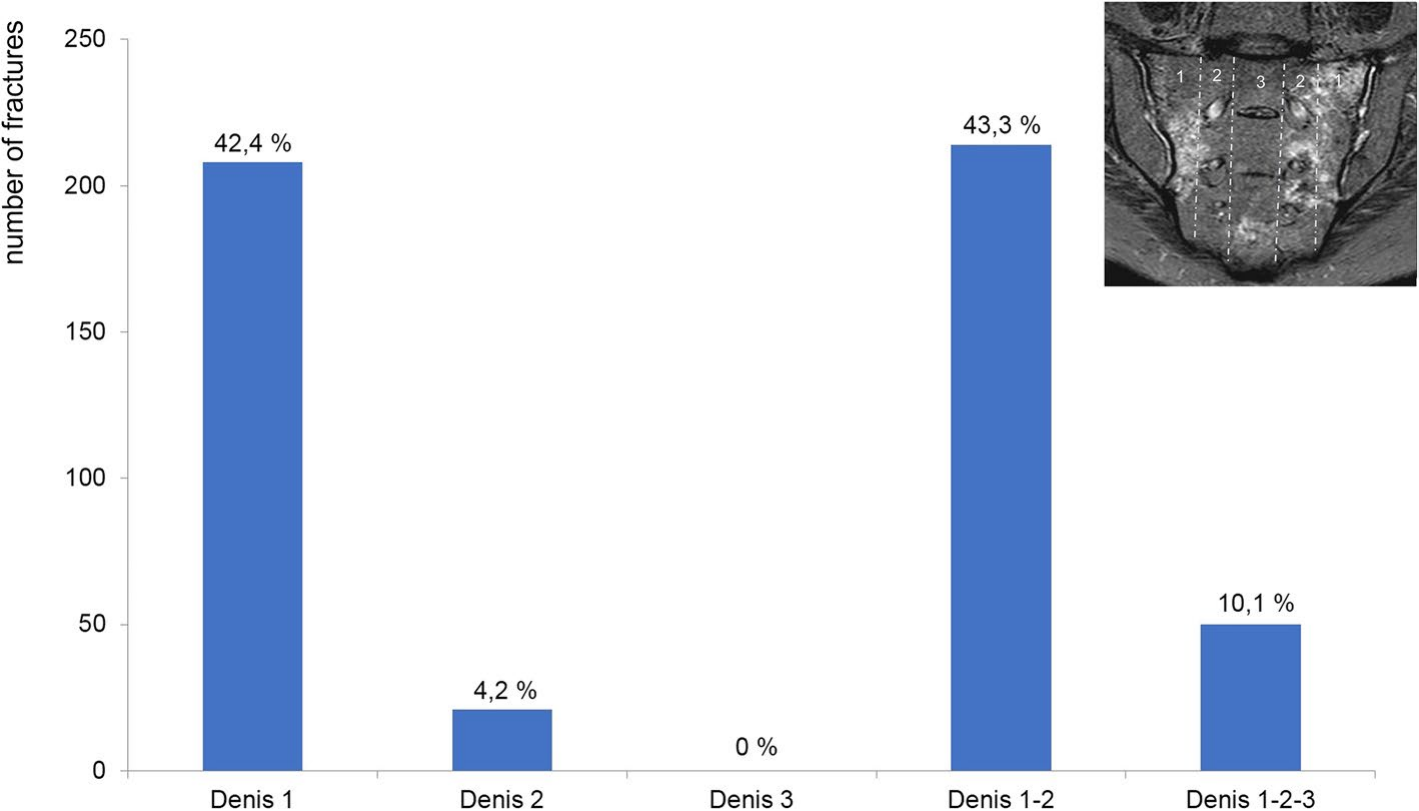
Frequency distribution of the fracture zones according to Denis et al. [24]. Image top right: Fracture zones marked on a semi-coronal MRI scan oblique to the sacrum. STIR weighting shows clear oedema on the right as an expression of a non-displaced fracture in the type 1 zone and on the left in the type 1 and 2 zones. A small area of oedema is also found in the caudal region of the type 3 zone

With a distribution of 208 out of 493 of the FFS, 42.4% were found to have a Denis type 1, 21 out of 493 or 4.2% had a Denis type 2, 0 out of 493 or 0% had a Denis type 3, 214 out of 493 or 43.3% had a Denis type 1 – 2, and 50 out of 493 or 10.1% had a Denis type 1 - 2 - 3 fracture zone (Fig. 3).

### Pain and mobility development

#### Conservative

**Fig. 4:**
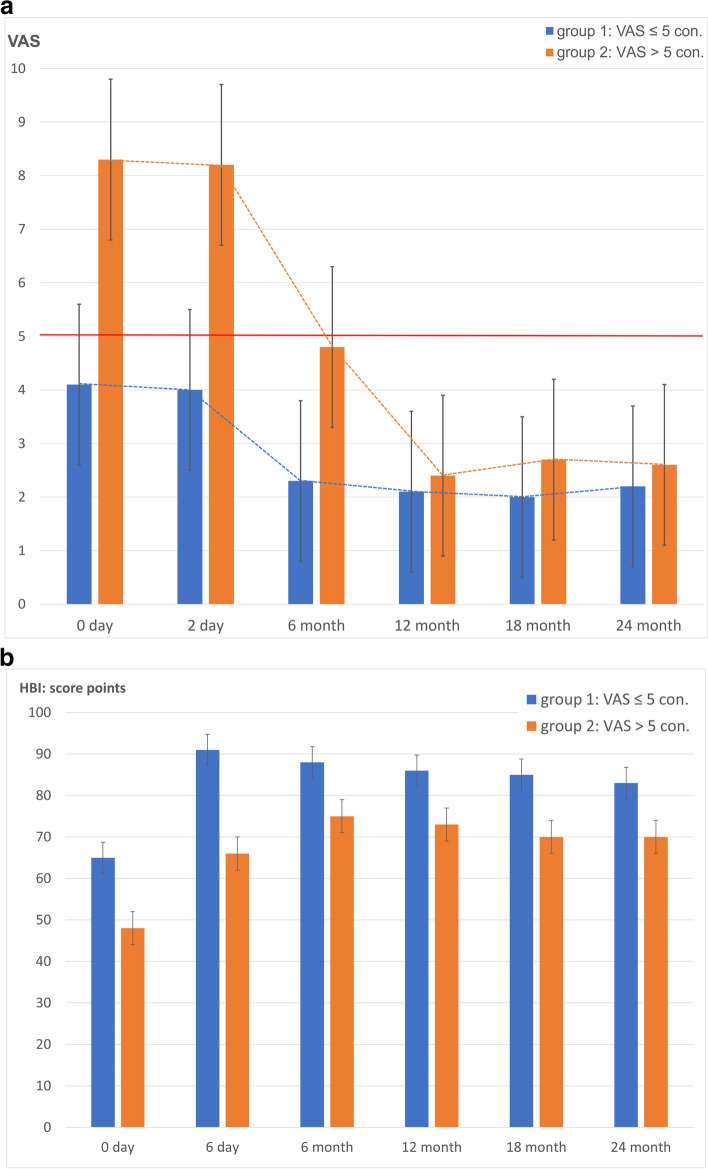
**a** Pain development of the conservatively treated patients over time. **b** Mobility development based on the HBI of conservatively treated patients

Consecutively, 50 patients with a pain level ≤ 5 (Group 1) and 100 patients with a pain level > 5 (Group 2) were included. Patients with a pain level of ≤5 benefited from the conservative therapy measures, whereby pain levels > 5 significantly delayed the development of mobility (Fig. 4 a and b).

A threshold for pain at 5 score points is marked in the graph by a solid horizontal line.

The patients in Group 2, starting from a high level of pain, only fell below a pain threshold of 5 score points after 6 months on average.

A total of 88 out of 114 (77.2%) patients could still be contacted after 24 months.

Comparison of the mean HBI values of patients with moderate (Group 1) and moderate to severe immobilising functional impairments (Group 2). The mean HBI of all patients was 55 +/− 15 at baseline, with a significant difference of 65 +/− 10 for Group 1 and 48 +/− 14 for Group 2, at *p* < 0.001. After 24 months, the scores increased to 76 +/− 13, with score points remaining significantly different (*p* < 0.05) between Group 1 and Group 2 at all-time points, with significantly lower scores for Group 2 patients.

**Fig. 5:**
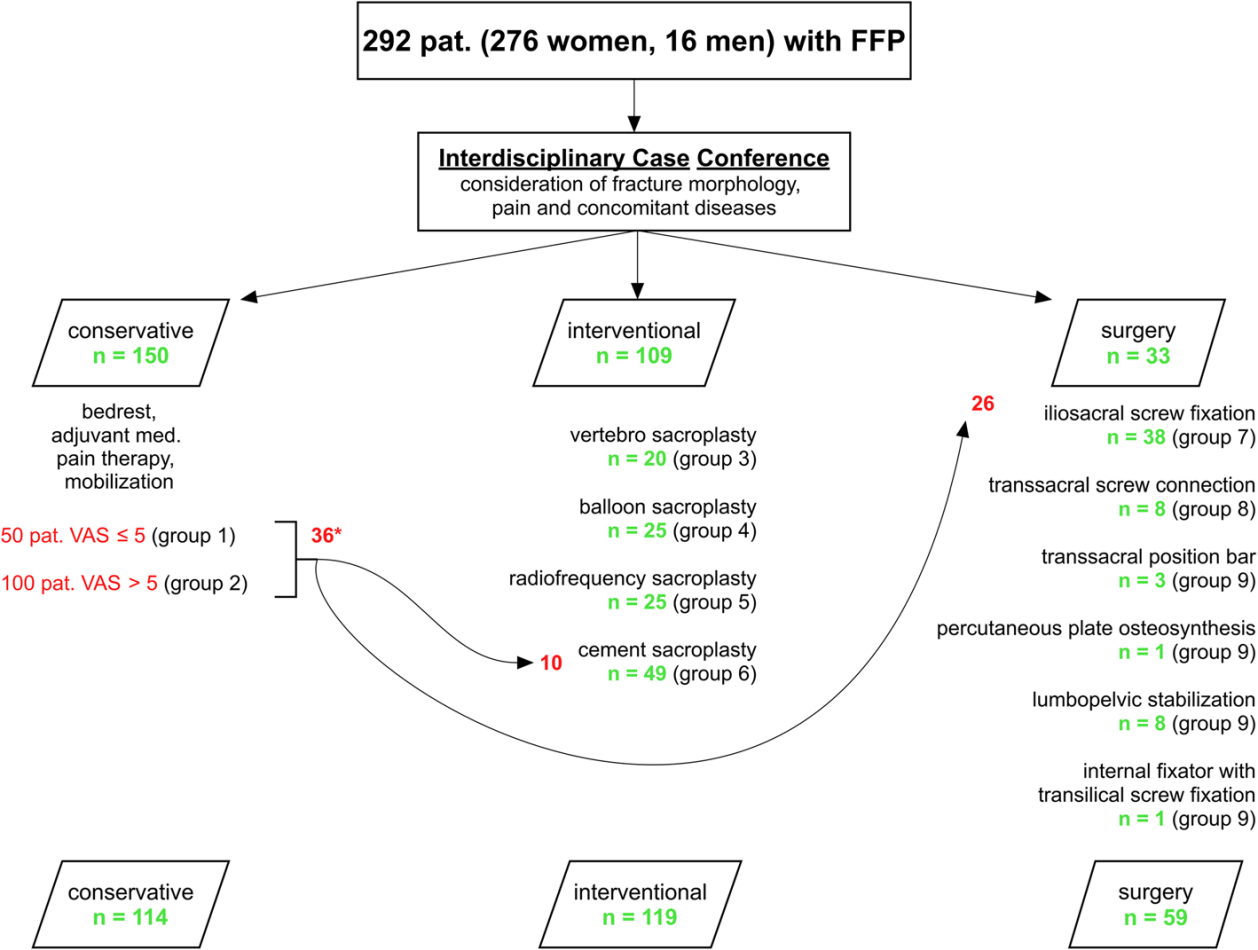
Illustration of the patient distribution among the different therapy options. 36* patients out of the conservative group underwent interventional (10) or surgical treatment (26) due to increased pain, pronounced immobility or increased fracture extension

Over the course of the study, 36 (4 Group I, 32 Group II) out of 150 (24%) patients were referred for interventional (10 out of 36) and osteosynthetic therapy (26 out of 36), due to increasing fracture extension, pain > 7 and pronounced immobility (Fig. 5). In the interventional group, 3/10 had a FFP IIa, 4/10 a FFP IIb and 3/10 a FFP IIc fracture. In the surgical group, 6/26 had a FFP IIb and 20/26 a FFP IIc fracture.

#### Interventional

**Fig. 6:**
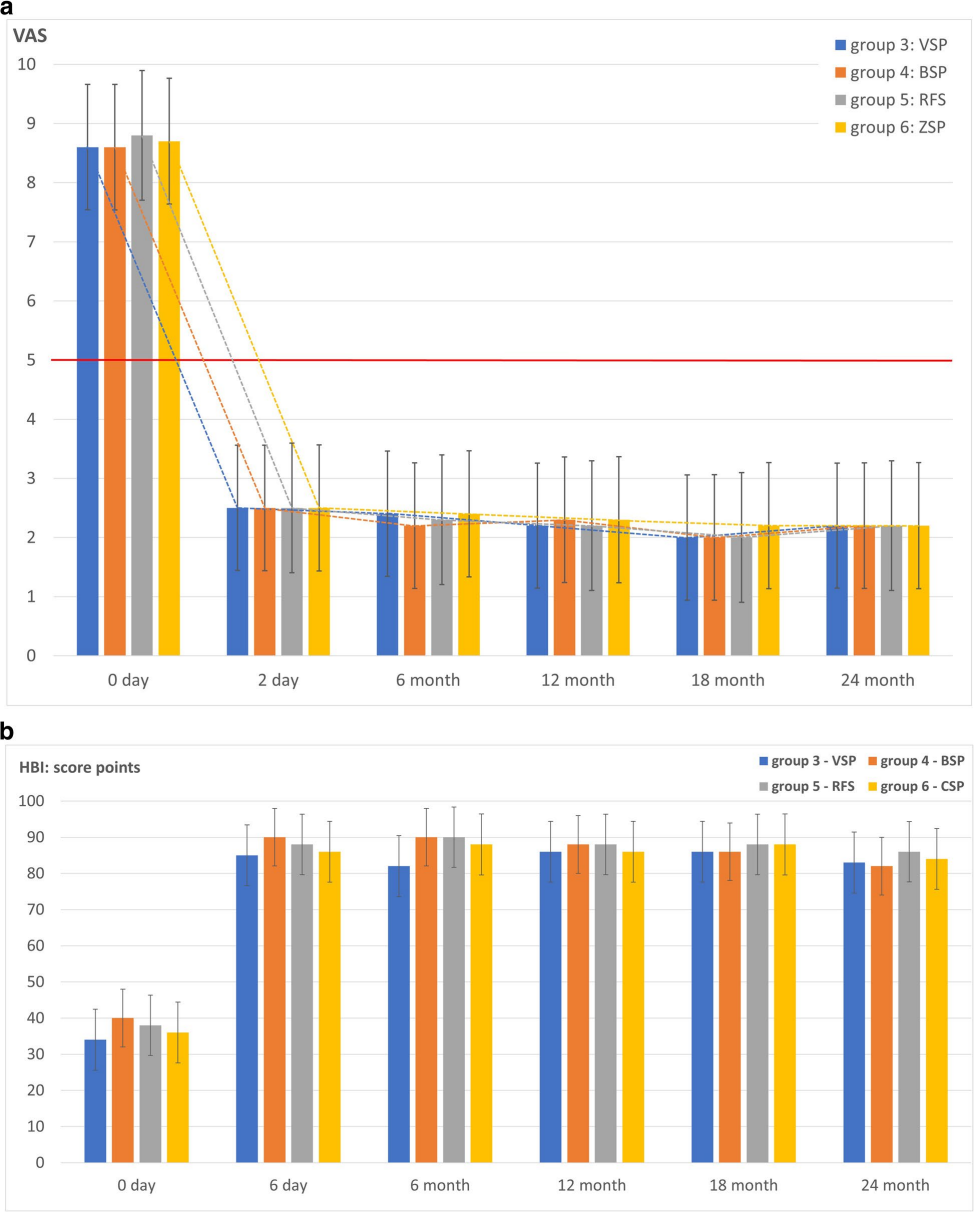
**a** Pain development of the interventionally treated patients over time. **b** HBI scores measured at different time-points in patients treated with sacroplasty

After sacroplasty, pain was reduced rapidly and significantly (*p* < 0.001), which quickly allowed a marked improvement in mobility, with no significant difference found between vertebro- (VSP), balloon (BSP), radiofrequency (RFS) and cement (CSP) sacroplasty (Fig. 6 a and b).

A threshold value for pain at 5 score points is marked in the graph by a solid horizontal line.

Patients in Groups 3 to 6 showed a significant (*p* < 0.001) reduction in pain as early as the second post-interventional day with a stable sustained effect over time, with no difference between groups.

A total of 109 out of 119 (91.6%) patients treated could still be contacted after 24 months.

The average HBI of all patients was 37 +/− 6 at baseline and 83 +/− 6 after 24 months. After 6 days, there was a significant (*p* < 0.001) improvement, with a sustained effect over 24 months. There was no significant difference between Groups 3 to 6 at *p* > 0.87.

A total of 119 patients underwent cement augmentation (Fig. 5). Cement leakage was found in 4 out of 20 patients (20%) after VSP (Group 3) and in 6 out of 49 patients (12.2%) after CSP (Group 4). None of the leaks were symptomatic. For BSP (Group 5) and RFS (Group 6) with 25 patients each, leakage was ruled out.

#### Osteosynthetic

The planned osteosyntheses of 59 patients with the following fracture morphology:

- FFP type II = Group 7: 2 FFP type II a, 3 FFP type II b and 9 FFP type II c;
- FFP type III = Group 8: 14 FFP type III c and.
- FFP type IV = Group 9: 26 FFP type IV b and 5 FFP type IV c

were carried out as planned (Fig. 5).

Iliosacral screw fixation was performed 38 times (with additional cement augmentation in 32 of 38), transsacral screw fixation 8 times, a transsacral positioning rod 3 times, percutaneous plate osteosynthesis once, lumbopelvic stabilisation 8, and an internal fixator with additional transiliac screw fixation once.

**Fig. 7:**
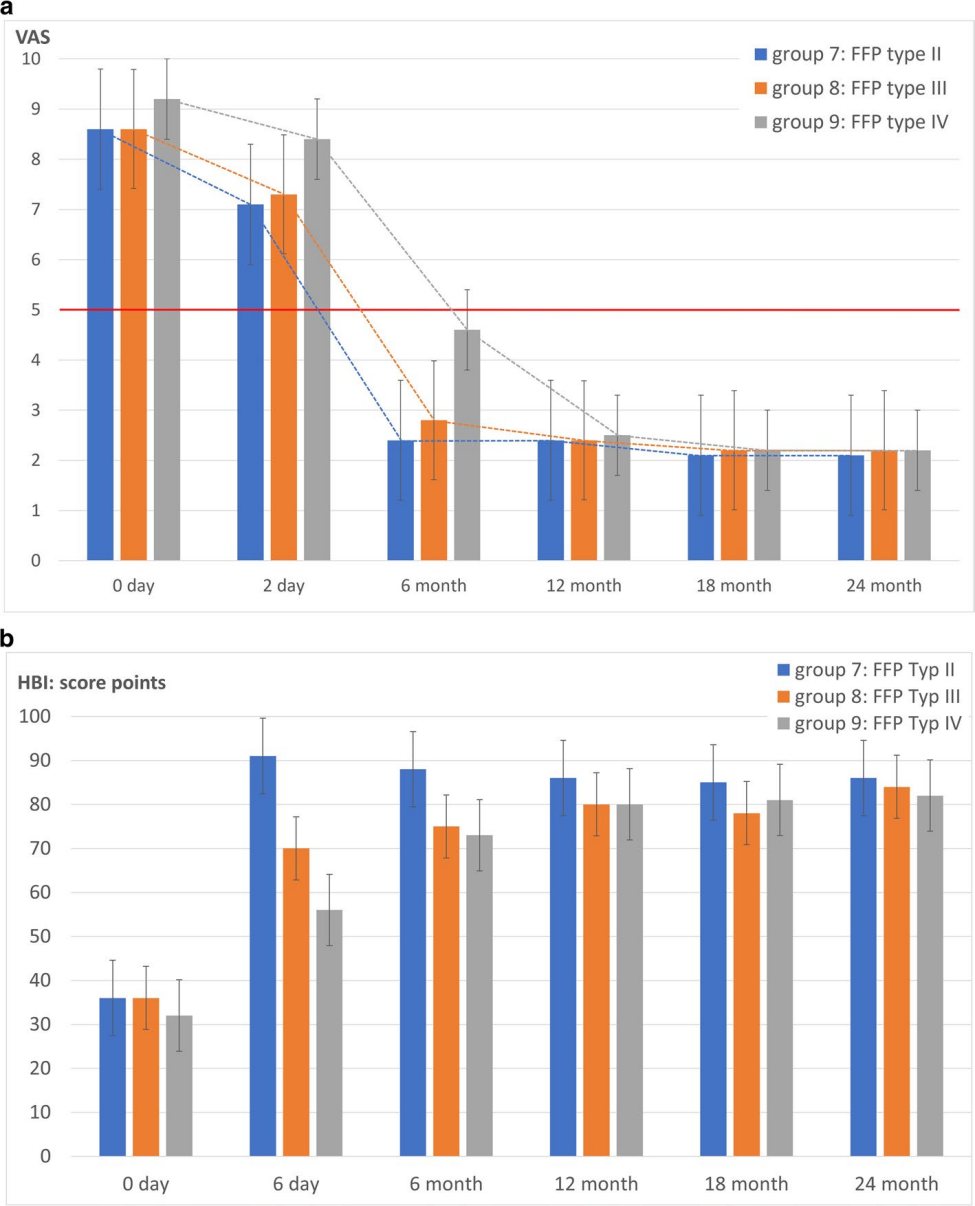
**a** Pain development of patients treated with osteosynthesis over time. **b** Mobility development based on the HBI of patients treated with osteosynthesis

In terms of pain reduction and mobilisation capacity, patients benefited from osteosynthesis, although more complex fracture types with lumbopelvic stabilisation performed required a longer period of recovery (Fig. 7 a and b).

A threshold value for pain at 5 score points is marked in the graph by a solid horizontal line.

Baseline pain is most pronounced in patients with more complex fractures (Group 9 - FFP type IV), with pain declining less rapidly over the postoperative course compared to Group 7 - FFP type II and Group 8 - FFP type III, the differences were not significant. After 12 months, the pain levels were more or less evened out at a low level. Forty five of 59 (76.3%) patients could still be contacted after 24 months.

The average HBI of all patients was 35 +/− 4 at baseline and 84 +/− 6 after 24 months, with no relevant difference between the groups at these points.

The patients in Group 9 - FFP type IV were significantly less mobile on postoperative day 6 than the patients in Group 7 - FFP type II and Group 8 - FFP type III.

Material loosening occurred in 8 of 59 patients, but this did not require revision.

There were no deaths during the hospital stay.

In the assessment of self-reliance, patients achieved an average of 76 score points after conservative therapy, 83 score points after sacroplasty and 84 score points after osteosynthesis at the end of 24 months.

However, of the 292 patients treated, only 81 patients (27.7%) achieved the same physical fitness as before the fracture event.

### Mortality

The mortality rate after 12 months was 21.7% for the conservative, 8.4% for the interventional and 13.6% for the surgical therapy group; the differences are significant. In patients in the conservative therapy group who were difficult to mobilise due to pain, the mortality rate increased to 24.3% (Table 2).

**Table 2:** Mortality in comparison of the therapy options

Therapy (Group)	12-month mortality (%)
Conservative	21.7
(1 and 2)	(18.4 and 24.3)
Interventional	8.4
(3; 4; 5 and 6)	(9.1; 8.0; 8.1 and 8.2)
Osteosynthetic	13.6
(7; 8 and 9)	(11.1; 10.5 and 16.1)

Group 1: Conservative therapy at a pain level ≤ 5

Group 2: Conservative therapy at a pain level > 5

Group 3: Cement augmentation by VSP

Group 4: Cement augmentation by BSP

Group 5: Cement augmentation by RFS

Group 6: Cement augmentation by CSP

Group 7: Osteosynthesis of FFP type II

Group 8: Osteosynthesis of FFP type III

Group 9: Osteosynthesis of FFP type IV

Over a period of 12 months after the start of therapy, the deceased could be clearly recorded.

The average percentages differed significantly between Groups 1 and 2 at *p* < 0.05. No significant difference was found between Groups 3 to 6 and Groups 7 to 9 at *p* > 0.83.

The conservatively treated patients showed a significant difference compared to the interventionally treated patients at *p* < 0.001 and compared to the osteosynthetic treated patients at *p* < 0.05, with an effect size of 0.87 and 0.74, respectively.

### Patient satisfaction

Subjective satisfaction with the therapies was best after sacroplasty at 12 and 24 months, followed by osteosynthesis and conservative measures (Table 3).

**Table 3:** Patient satisfaction, for which the pain development from Figs. 4, 5 and 6a as well as the mobility development from Figs. 4, 5 and 6b were taken into account. Post-therapeutic, persistent pain > 5, as in Groups 2 and 9, blocks rapid mobilisation and leads to moderate to poor satisfaction

Group	Pain reduction	Development of mobility and self-reliance	Subjective satisfaction
1	slow, acceptable	moderate	moderate
2	slow, inacceptable	moderate to poor	poor
3	rapid, good	marked	good
4	rapid, good	marked	good
5	rapid, good	marked	good
6	rapid, good	marked	good
7	rapid, good	marked	good
8	rapid, good	marked	good
9	delayed, acceptable	moderate	moderate

Group 1: Conservative therapy at a pain level ≤ 5

Group 2: Conservative therapy at a pain level > 5

Group 3: Cement augmentation by VSP

Group 4: Cement augmentation by BSP

Group 5: Cement augmentation by RFS

Group 6: Cement augmentation by CSP

Group 7: Osteosynthesis of FFP type II

Group 8: Osteosynthesis of FFP type III

Group 9: Osteosynthesis of FFP type IV

### Vitamin D and BMD

All patients had a pronounced vitamin D deficiency and manifest osteoporosis.

### Vitamin D

**Fig. 8:**
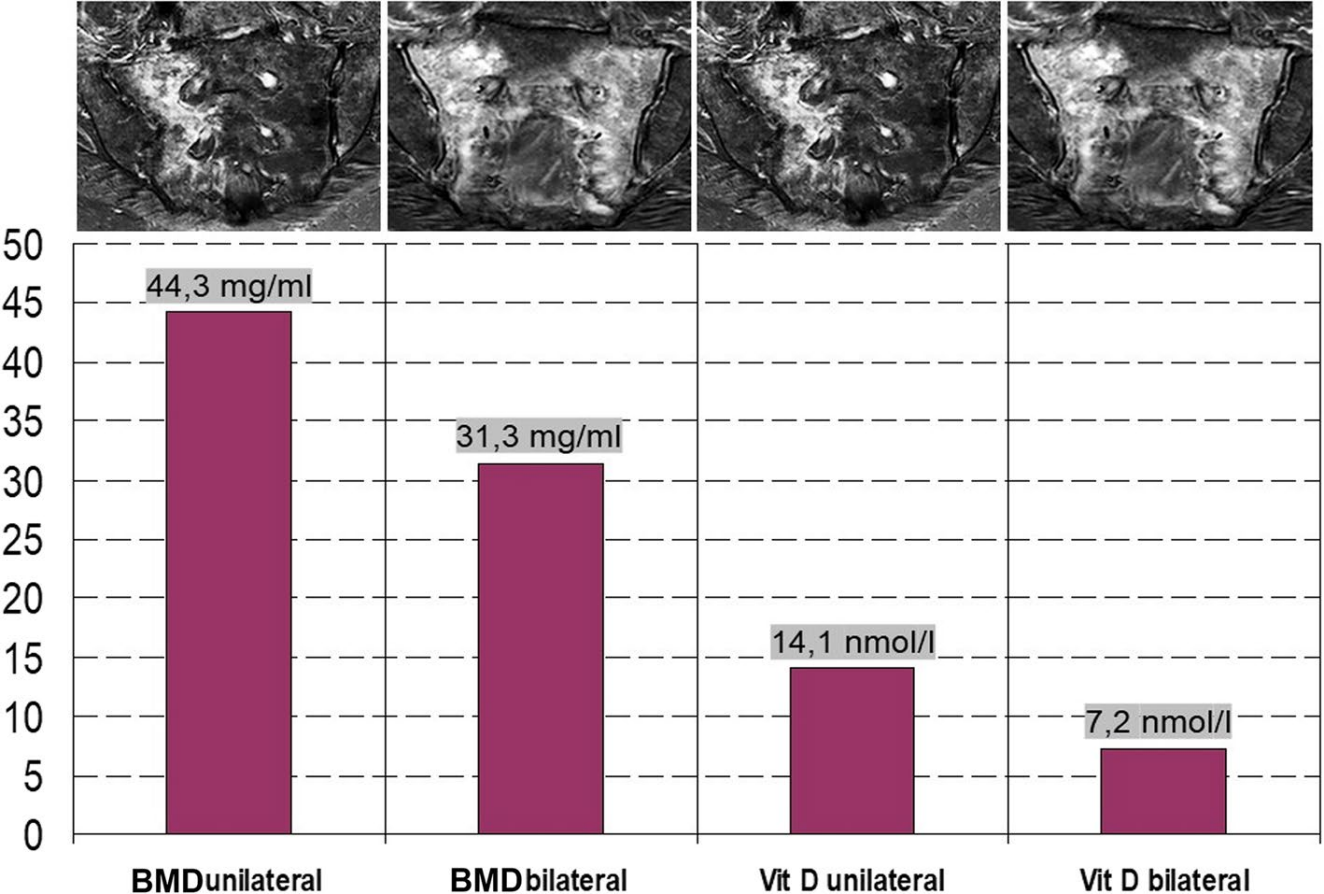
Comparison of bone mineral density (BMD) values of the lumbar spine and vitamin D values in unilateral and bilateral FFS

The vitamin D level was significantly (*p* < 0.001) lower than 30 nmol/l ≙ 12 ng/ml in all patients. Vitamin D levels were 8 - 28 (Ø 14.1) nmol/l ≙ 3.2 - 11.2 (Ø 5.6) ng/ml in unilateral fractures and 0 - 18 (Ø 7.2) nmol/l ≙ 0 - 7.2 (Ø 2.9) ng/ml in bilateral, more complex fractures (Fig. 8), the difference in mean vitamin D levels being significant (*p* < 0.05).

### BMD

The BMD was 12-74 (Ø 44.3) mg/ml in the patients with a unilateral fracture and 2 - 54 (Ø 31.3) mg/ml in the patients with a bilateral fracture (Fig. 8), the difference in mean BMC values being significant (*p* < 0.05).

### Disease profile

In 128 of 292 (43.8%) patients in total, at least one previous sintering fracture was found in the thoracic and lumbar spine. Other osteoporosis-associated fractures such as distal radius, proximal humerus, femoral neck, rib and sternal fractures were found in 142 of the 292 (48.6%) patients. Hypocalcaemia was found in 35% and secondary hyperparathyroidism in 48% of all patients. Additional lung disease was found in 23.6%, cardiovascular disease in 45.2%, hypertension in 77.1%, renal insufficiency in 34.2%, diabetes mellitus type II in 72.8%, PAOD in 70.3% and obesity in 60.2% of all patients. A varying degree of nicotine consumption was reported by 48.3% of all patients.

## Discussion

Up to our knowledge, this is the first study which compared the outcome of three different therapy options for the treatment of FFS.

### Clinical aspects and profile of the patients

As in other publications, the risk factors confirmed in our patient population with sacral insufficiency fractures were advanced age [1, 5, 12, 30], female gender [9, 12, 31], drastic vitamin D deficiency [32, 33] and osteoporosis [30, 33, 34]. As in Maier et al. [33], where the measurement was done by DXA, all our patients also showed osteoporosis in the osteodensitometric measurement by QCT. The BMD values of the QCT measurement on the axial skeleton were clearly below the threshold for osteoporosis of 80 mg/ml [35], whereby the significantly lowest values were found in complex sacral fractures, with an average of 31.3 mg/ml. The additional sintering fractures of 43.8% of all patients in the axial skeleton and fractures of 48.6% of all patients in the peripheral skeletal region support the presence of clinically manifest osteoporosis in the patient collective.

The fracture types found most frequently were a Denis 1 in 42.4% and Denis 1-2 in 43.3% and an FFP II in 85.7%, this distribution also being found by other working groups [26, 36]. With generally preceding fracture dynamics [37, 38], which were shown by varying degrees of oedema and sclerosis of individual fracture zones in contralateral comparison, bilateral sacral fractures were clearly predominant, with 68.8% in 292 patients. Since bilateral fractures are more unstable [38] and allow more micromovements in the fracture zones, this also explains the high level of pain and the pain-related immobility of our patients, which was comparable to the findings of a previous study [34]. The number and percentage distribution of additional concomitant diseases are similar to those found by Maier et al. [33].

### Outcome in the comparison of conservative, interventional and osteosynthetic therapies, considering pain, mobility and patient satisfaction

#### Conservative

Patients with baseline pain ≤5 (Group 1) on the VAS experienced a reduction in pain (Fig. 4a) and an increase in mobility and self-reliance (Fig. 4b) at an acceptable level with moderate patient satisfaction (Table 3). In the patients with initial pain > 5 (Group 2), there was a clearly delayed reduction in pain (Fig. 4a) and a moderate improvement in self-reliance (Fig. 4b) accompanied by poor satisfaction with the situation after the fracture event (Table 3). Maier et al. [33] also found a significant loss of self-reliance under conservative therapy after a fracture event. If there was no response to therapy, 36 out of 100 patients were transferred to the interventional or osteosynthetic therapy group, a procedure also recommended by Josten & Höch [37]. Nuber et al. [39] recommend osteosynthesis if conservative therapy fails, while others prefer sacroplasty [34, 40]. Especially if fracture progression then occurs with increasing instability, osteosynthesis should be performed at an early stage [37, 41].

#### Interventional

In comparison, after sacroplasty (Group 3 = VSP, Group 4 = BSP, Group 5 = RFS and Group 6 = CSP), the patients experienced a rapid and significant reduction in pain (Fig. 6a) with a rapid and significant improvement in self-reliance (Fig. 6b) and good patient satisfaction (Table 3) without any difference between the groups, as is to be expected given the same mechanism of action [26, 42]. The mechanism of action is based on stabilisation and minimisation of micromovements by the PMMA cement plug inserted into the fracture zone [43–45], which leads to a reduction in pain. Rapid, significant and sustained pain reduction is the greatest benefit for patients after sacroplasty. This has been found by many studies [26, 34, 42, 46–52] and is supported by comparable results from multicentre studies [53, 54], systematic reviews and meta-analyses [8, 55–59]. Schwetje et al. [51] were also able to show a significant increase in mobility after BSP in the absence of an effect under conservative therapy.

#### Osteosynthetic

Starting from a high level of pain, there was a significant overall reduction in pain after the 6th postoperative day, with patients with FFP type II and FFP type III showing a clearer reduction in pain than patients with FFP type IV (Fig. 7a), which was also reflected in the mobility development based on the HBI (Fig. 7b). The minimally invasive osteosyntheses performed in our patients led to primary stability with the possibility of full weight-bearing and pain-oriented mobilisation. Depending on the extent of fracture classification and assessed instability, as in other study groups [27, 37, 60, 61], iliosacral screw fixation was used most frequently, followed by transsacral screw fixation, transsacral positioning rod, plate osteosynthesis, lumbopelvic stabilisation and internal fixator with transiliac screw fixation. The iliosacral screw osteosynthesis leads to good compression and thus stabilisation, especially in vertically running fracture zones [61, 62]. This can be used to achieve a significant reduction in pain, as in our patients [63]. Due to the pronounced osteopenic bone texture, additional cement augmentation was performed in 32 of 38 iliosacral screws inserted. This procedure appears to be safe, promises greater stability and minimises the risk of complications [64, 65]. As in our case, Höch et al. [64] found a significant and sustained reduction in pain postoperatively in older, osteoporotic patients. Biomechanically, cement augmentation also reduces screw loosening in osteoporotic bone, supporting this approach [66]. In 8 out of 59 osteosyntheses, a transsacral screw fixation was performed, which allows an alternative to the cemented iliosacral screw due to a bilateral anchorage of the screw in the ilium [67], whereby no difference was found in the outcome in our patients. In 8 of 59 patients with a bilateral sacral fracture corresponding to an FFP type IV b without major dislocations of the sacral fracture fragments, good primary stability could be achieved by minimally invasive lumbopelvic stabilisation, as also presented by other working groups [60, 63, 68, 69]. With regard to pain reduction, mobility development and subjective satisfaction, however, an acceptable clinical improvement was only seen over the postoperative course after 6 to 12 months, comparable to the results of Mendel et al. [69].

### Outcome considering complications and mortality

With regard to pain reduction and mobility development, patients with baseline pain > 5 on the VAS (Group 2) showed the worst development under conservative therapy, which was already found to be comparable elsewhere [34]. Patients with baseline pain < 5 on the VAS (Group 1) were better mobilised and benefited from conservative therapy [11, 70]. Concomitant diseases such as phlebothrombosis with pulmonary artery embolism, pneumonia and urosepsis were highest in patients with poor mobility development and primarily affected Group 2. After sacroplasty (Groups 3 - 6) and osteosynthesis (Groups 7 - 9) these were less frequent in percentage terms.

A comparison of the mortality rates yielding significant effects appears to be possible for a time span of 12 months after the start of therapy, and they can be reliably assigned to the different therapies, see Table 2.

A mortality rate of 21.7% in the conservative group (average value from Groups 1 and 2) was also found by other research groups [33, 34, 71, 72]. In patients who are difficult to mobilise due to severe pain, the 12-month mortality rate increases under conservative measures to 24.3%.

Patients in Groups 3-6 benefit considerably from the significant, short-term reduction in pain and the resulting rapid mobilisation after sacroplasty; the 12-month mortality rate was 8.4% on average. A similar tendency was already shown in a preliminary study comparing conservative therapy with a mortality of 23.5% against BSP with a mortality of 3% after 12 months [34]. There was no significant difference between VSP, BSP, RFS and CSP.

Patients in Groups 7-9 also seem to benefit significantly from surgery compared to conservative therapy, with an average 12-month mortality of 13.6%, despite an initial situation with more complex fractures, a higher pain level and poorer HBI. This is also in line with the results of Bible et al. [73], who were able to show that after conservative fracture treatment, mortality after one year was twice as high (15%) as after surgical stabilisation. Hoech et al. [74] found a significantly higher survival rate for surgically treated patients of 21% compared to conservatively treated patients over a two-year period. With a comparable distribution of FFP types, Rommens et al. [75] also found a lower mortality in surgically treated patients. The one-year mortality rate of surgically treated patients with FFP types II-IV was halved in comparison with conservatively treated patients [76]. As in our case, surgically treated patients benefit from minimally invasive percutaneous procedures in terms of complication rate and mortality [77].

## Strengths and limitations

The benefit of our study is the multicenter design and the comparison of three different treatment modalities for addressing FFS with outcome measurements over a period of 24 months.

Limitations are the retrospective study design, the treatment of different fracture types and a bias due to the selection of the type of treatment (surgical versus interventional).

## Conclusions

**Fig. 9:**
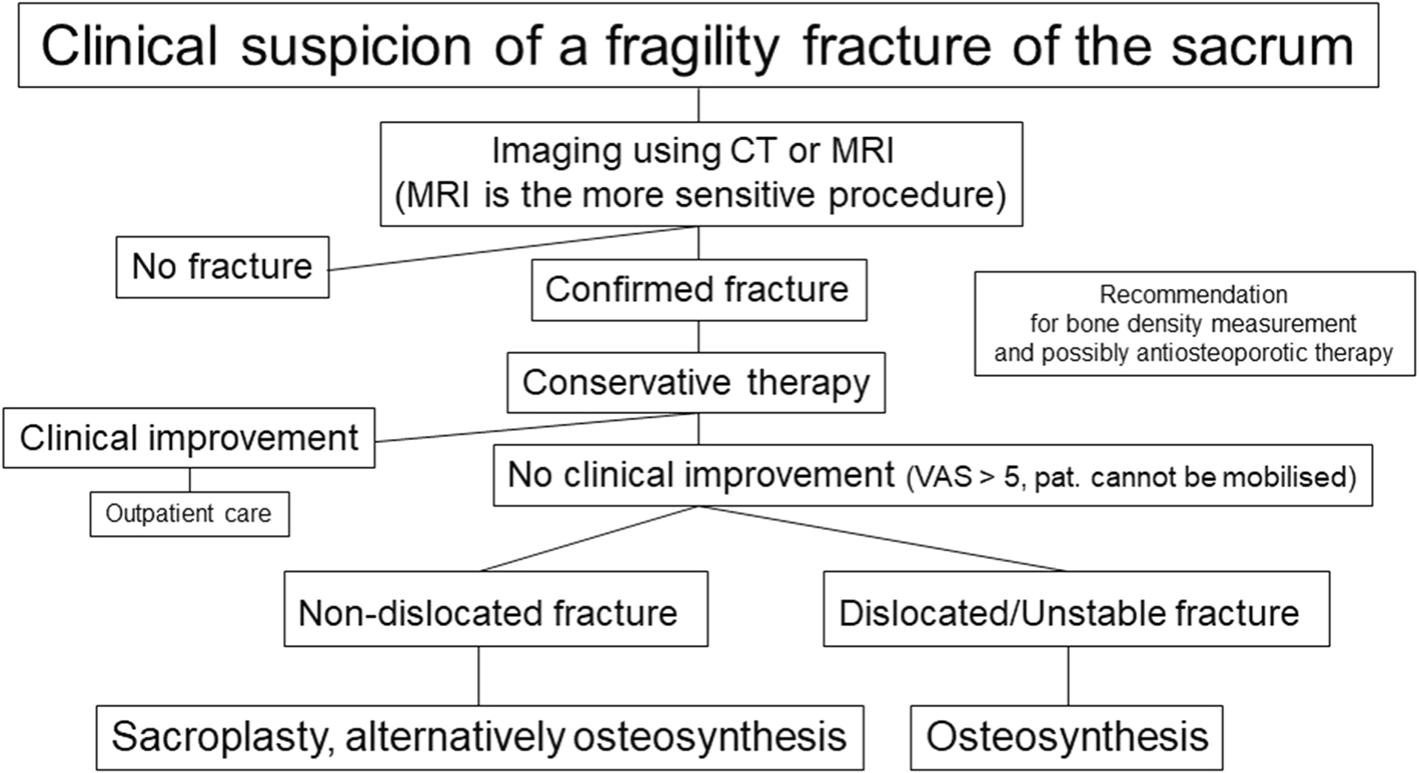
Staged plan for the therapeutic approach in the treatment of FFS. Different interventional procedures and techniques are described for sacroplasty by Andresen et al. [26] and surgical procedures for osteosynthesis by Oberkircher et al. [27]

In order to avoid consecutive disablement, prompt diagnostics and multimodal, interdisciplinary therapy are necessary in patients with FFS. Patients with a low level of pain can be treated conservatively [11]. Patients with severe pain and non-dislocated fractures benefit from cement augmentation effectively and sustainably, whereby different methods are available [26]. Patients with disabling pain and unstable fractures should be treated osteosynthetically as early as possible [37]. Regardless of whether a conservative, interventional or osteosynthetic therapy is chosen, a guideline-compliant antiosteoporotic treatment [29] is necessary, whereby to accelerate fracture healing an osteoanabolic treatment [78] should be chosen. A diagnostic and therapeutic algorithm for managing FFS is shown in Fig. 9.

## Data Availability

The datasets used and analysed during the current study available from the corresponding author on reasonable request.

## Abbreviations

ANOVA: Analysis of variance
CSP: Cement sacroplasty
BMD: Bone mineral density
BSP: Balloon sacroplasty
CT: Computed tomography
FFS: Fragility fractures of the sacrum
FFP: Fragility fractures of the pelvis
HBI: Hamburg Barthel index
MRI: Magnetic resonance imaging
PAOD: Peripheral arterial occlusive disease
PMMA: Polymethyl methacrylate
QCT: Quantitative computed tomography
RFS: Radiofrequency sacroplasty
STIR: Short-Tau inversion recovery
VAS: Visual analogue scale
VSP: Vertebro sacroplasty
WHO: World Health Organization
